# Starch-based thickening in infant formula: *in vitro* study of behavior in the bottle and under gastric conditions

**DOI:** 10.3389/fnut.2026.1803756

**Published:** 2026-04-10

**Authors:** Charyssa De Smul, Josephine Braekman, Lotte De Wever, Valérie Vanhoorne, Eline Tommelein

**Affiliations:** 1 Faculty of Medicine and Health Sciences, Ghent University, Ghent, Belgium; 2 Laboratory of Pharmaceutical Technology, Department of Pharmaceutics, Ghent University, Ghent, Belgium; 3 Department of Pharmaceutical and Pharmacological Sciences, Experimental Pharmacology, Vrije Universiteit Brussel, Jette, Belgium

**Keywords:** infant formula preparation, reflux, regurgitation, starch, viscosity

## Abstract

**Introduction:**

The addition of starch-based thickening agents to increase the viscosity of infant formula is common practice in primary care. However, this strategy lacks standardized preparation steps and a benchmark for therapeutic effect. Therefore, this study aims: (1) to determine the optimal preparation method for infant formulas (pre-)thickened with starch; (2) to evaluate their viscosity in the bottle; (3) to assess their viscosity under simulated gastric conditions; and (4) to estimate the caloric contribution of externally added starch-based thickeners to standard infant formulas.

**Methods:**

We performed *in vitro* rheological measurements in a standard formula externally thickened with rice flour [SFRF] and a commercial formula pre-thickened with potato starch [IFPS], using a rotational rheometer under bottle-simulated and gastric-simulated conditions. Experiments were performed across different pH levels (pH 1, pH 4 and pH 7) and at multiple time points (5–60 min). Gastric simulation included the addition of artificial saliva and simulated gastric fluid. A descriptive analysis was performed.

**Results:**

The results show that infant formulas containing starch, whether inherently pre-thickened or prepared with added starch-based thickeners, require vigorous shaking to ensure complete solubilization of starch granules. Preparation at 37 °C further supports more proper dispersion over preparation at 20 °C. Standard formulas thickened with rice flour demonstrate limited stability, with a marked decline in viscosity after 20 min at 20 °C, indicating they should be prepared and consumed promptly. In contrast, formulas pre-thickened with potato starch reach their peak viscosity only after approximately 30 min. Nevertheless, when exposed to simulated gastric conditions, the elevated viscosities observed in the bottle for both types are not maintained but rather comparable to those of standard, non-thickened formulas. Furthermore, adding starch-based thickeners substantially elevates the caloric content of infant feeds.

**Conclusion:**

Our findings suggest that SFRF should be administered within 20 min of preparation and IFPS must be shaken *vigorously*. The present study highlights potential concerns regarding the *in vivo* relevance of starch-based thickening for increasing gastric viscosity, although additional studies are warranted to validate these observations.

## Highlights

What is known

- Addition of starch-based thickening agents to increase viscosity of infant formula is common practice.
- The evidence to support the use of thickened formulas over standard formulas is limited.
- No serious adverse effects have been reported with the use of thickening agents.

What is new

- When aiming for high viscosity in the bottle, rice flour appears preferential over potato starch.
- Ideal preparation protocols differ between types of starch, i.e., potato or rice.
- Under simulated gastric conditions, viscosities of starch-thickened and non-thickened formulas are comparable, questioning their clinical efficacy.

## 1 Introduction

Thickening agents are frequently used in or added to infant formulas to increase viscosity, primarily to manage reflux, regurgitation, or dysphagia (1). Thickening agents fall into three categories: digestible starch-based thickeners made from rice, potato or corn; indigestible gum-based thickeners, including carob bean gum [CBG] and xanthan gum; and indigestible cellulose-based thickeners, such as sodium carboxymethylcellulose [NaCMC] (1–3). While some infant formulas come pre-thickened by manufacturers, many thickening agents are also available as standalone products that can be added to standard infant formulas during preparation.

Some limited evidence supports the use of thickened formulas over standard formulas, showing reductions in regurgitation or fewer episodes of regurgitation and vomiting per day (4). The use of thickened formulas, as compared to a control treatment, was also associated with a reduction in crying, dysphagia and regurgitation-related symptoms (e.g., irritability, coughing, choking and night awakening) (4). However, thickened formulas seem not to affect reflux index, the frequency of acid reflux episodes, or reflux episodes lasting over 5 min (4). Aside from a potential increased risk of necrotizing enterocolitis (NEC) associated with xanthan gum, no serious adverse effects have been reported with the use of thickening agents. Concerns nevertheless remain about increased energy expenditure during feeding with heavily thickened formulas, as well as potential impact on weight gain with starch-based thickeners (1, 5). Four randomized controlled trials involving 265 infants have indeed demonstrated that starch-thickened formulas lead to a statistically significant increase in weight gain compared to a reference formula (Weighted mean difference of 3.7 g/day [95% CI: 1.55 to 5.80]) (4).

Assessing the clinical effects of viscosity-enhancing agents *in vivo* is limited by ethical and practical constraints. At the same time, the technical demands of *in vitro* rheological testing also hinder the breadth of research on the effectiveness of thickening agents. To date, the study by Prakash et al. (6) remains the only *in vitro* investigation examining the flow behavior of infant formula in a simulated digestive environment. Within the broader field of clinical nutrition, viscosity modification can also be standardized using frameworks such as the International Dysphagia Diet Standardization Initiative (IDDSI), which classifies liquids and foods into defined texture levels using simple, clinically applicable testing methods. Earlier systems, such as the National Dysphagia Diet, similarly categorized fluid consistencies, although these frameworks are primarily developed for dysphagia management in older populations and are not specifically validated for infant feeding. As a result, several important clinical questions remain unanswered—such as the minimum viscosity required to achieve a therapeutic effect, whether this threshold varies by underlying condition (e.g., dysphagia, reflux, or regurgitation), and which thickening agent may be most appropriate in each context.

Moreover, there is a lack of research on the practical application of thickening agents in infant formulas, despite each type having distinct preparation requirements and characteristics that may influence its appropriateness for both the infant and their caregivers. For instance, formulas thickened with CBG or NaCMC show a steep increase in viscosity with even small concentration adjustments, making their preparation process prone to error (3). CBG also requires approximately 30 min. of standing time to reach final thickness (3, 7). Furthermore, NaCMC can considerably contribute to sodium intake in infants (3). For starch-based thickeners, there is no information on their optimal preparation method apart from the fact that they are unsuitable for use with human milk due to the presence of amylase (8, 9).

To address these knowledge gaps, the present study aims to investigate the viscosity behavior of starch-thickening of infant formulas over time, across varying temperatures and pH levels, under three simulated conditions: a bottle simulation, a simple gastric simulation, and an advanced gastric simulation. The study is centered around whether the viscosity-enhancing effects of starch-based thickeners observed in the bottle are maintained under gastric conditions. By examining this, we aim to clarify the clinical relevance of these thickening strategies for managing reflux, regurgitation or dysphagia in infants. Two different infant formulas are investigated: a standard formula externally thickened with rice flour (SFRF) and a commercial formula pre-thickened with potato starch (IFPS). This study aimed (1) to determine the optimal preparation method for infant formulas (pre-)thickened with starch; (2) to evaluate the viscosity behavior of SFRF and IFPS in the bottle; (3) to assess their viscosity under simulated gastric conditions; and (4) to estimate the caloric contribution of externally added starch-based thickeners to standard infant formulas.

## 2 Methods

### 2.1 Infant formulas, thickening agents and reference values

A standard infant formula (Nutrilon® Profutura 1, Nutricia—SF) was chosen for this study due to its unmodified composition, not tailored to any specific clinical condition. We prospectively assessed the viscosity of this formula when supplemented with rice flour (Olvarit Rice Flour 4 + Months, Nutricia®—SFRF), and also evaluated a commercially available pre-thickened formula containing potato starch (NAN® Verzadiging 1, Nestlé—IFPS). Since this research involves preclinical first-hand experiments, two representative examples of starch-based thickening in practice were selected. This selection process was guided by literature search focussed on thickening strategies in the Belgian pediatric practice. The nutritional profiles are provided in Table 1. The experiments were conducted from July until September 2024. The viscosity of olive oil and the SF were included as references to facilitate ease of interpretation and provide a familiar benchmark for comparing the thickness of the tested formulas. It should be noted that no dilution was applied for these references. The reference viscosities, as established during a previous analysis in our lab, represent the mean of a triplicate of rheological analyses conducted at pH 7 and 37 °C, and equal 34 mPa·s ± 0.003 mPa·s for olive oil and 2 mPa·s ± 0.138 mPa·s for the SF (3).

**Table 1:** Nutrient composition of selected infant formulas (Nutrilon Profutura®, Nutricia and NAN® Verzadiging 1, Nestlé) prepared per 100 mL of mineralized bottled water.

Sample type	Thickening mechanism	Caloric value (kcal/100 mL)	Protein (g/100 mL)(whey/casein ratio)/Protein hydrolysation	Carbohydrates (g/100 mL; % starch of total carbohydrate content, carbohydrate source)	Lipids (g/100 mL)	Fibers (g/100 mL; %GOS, %FOS)
SF	None	66	1.3 (50/50) / No	7.3 (0%, NA)	3.4	0.7 (69, 11%)
Rice flour	2.5 g/100 mL	9.6	0.2	2.1 (85.6%, Rice)	0.03	0.01
SFRF	Rice flour/SF ratio: 0.33 g/g	75.6	1.5 (50/50) / No	9.4 (21%, Rice)	3.4	0.7 (69, 11%)
IFPS	Potato Starch	67	1.2 (70/30) / No	7 (29%, Potato)	3.6	0.26 (NA)

GOS, galacto-oligosaccharides; FOS, fructo-oligosaccharides; NA, not applicable; SF, standard infant formula, commercially available as Nutrilon Profutura®, Nutricia; rice flour: commercially available as Olvarit®; SFRF: standard infant formula thickened with rice flour; IFPS: pre-thickened infant formula with potato starch, commercially available as NAN® Verzadiging 1, Nestlé.

### 2.2 Sample preparation

For the SF, a 50 mL portion was prepared in a 250 mL round-bottom flask set in a thermostated oil bath on a magnetic stirring plate. Once 50 mL of mineralized bottled water (Chaudfontaine®) reached 37 °C, the amount recommended by the supplier of formula powder (7.667 g/50 mL) was added. The SF sample was then *moderately* shaken by hand for 20 s, mimicking routine daily preparation in a real-world context before being returned to continuous magnetic stirring at 300 rpm. Upon preparation, all samples demonstrated pH values between 6.52 and 7.54.

For the SFRF sample, 100 mL of Chaudfontaine® water was heated to 37 °C (±2 °C), after which formula powder and thickening agent—both corresponding to only 50 mL of water—were added under continuous stirring. The sample was thereupon *moderately* shaken before being returned to continuous stirring. Supplier-recommended concentrations were 15.333 g/100 mL for SF and 5.000 g/100 mL for the thickening agent. The concentrations used in the study were 7.667 g/100 mL for SF and 2.500 g/100 mL for the thickener. This 1:1 dilution (formula-to-water) was applied to allow comparison with samples prepared for the advanced gastric environment simulation, which included 50 mL of formula and 50 mL of enzymatic solution (see “advanced simulation protocol”). The latter takes into account that the prepared formula is diluted in the lumen of the stomach. Using identical dilutions in the bottle simulation and advanced gastric environment simulation allowed a direct comparison between the simulated conditions to evaluate the impact of the simulated gastric environment on the apparent viscosity.

**Figure 1:**
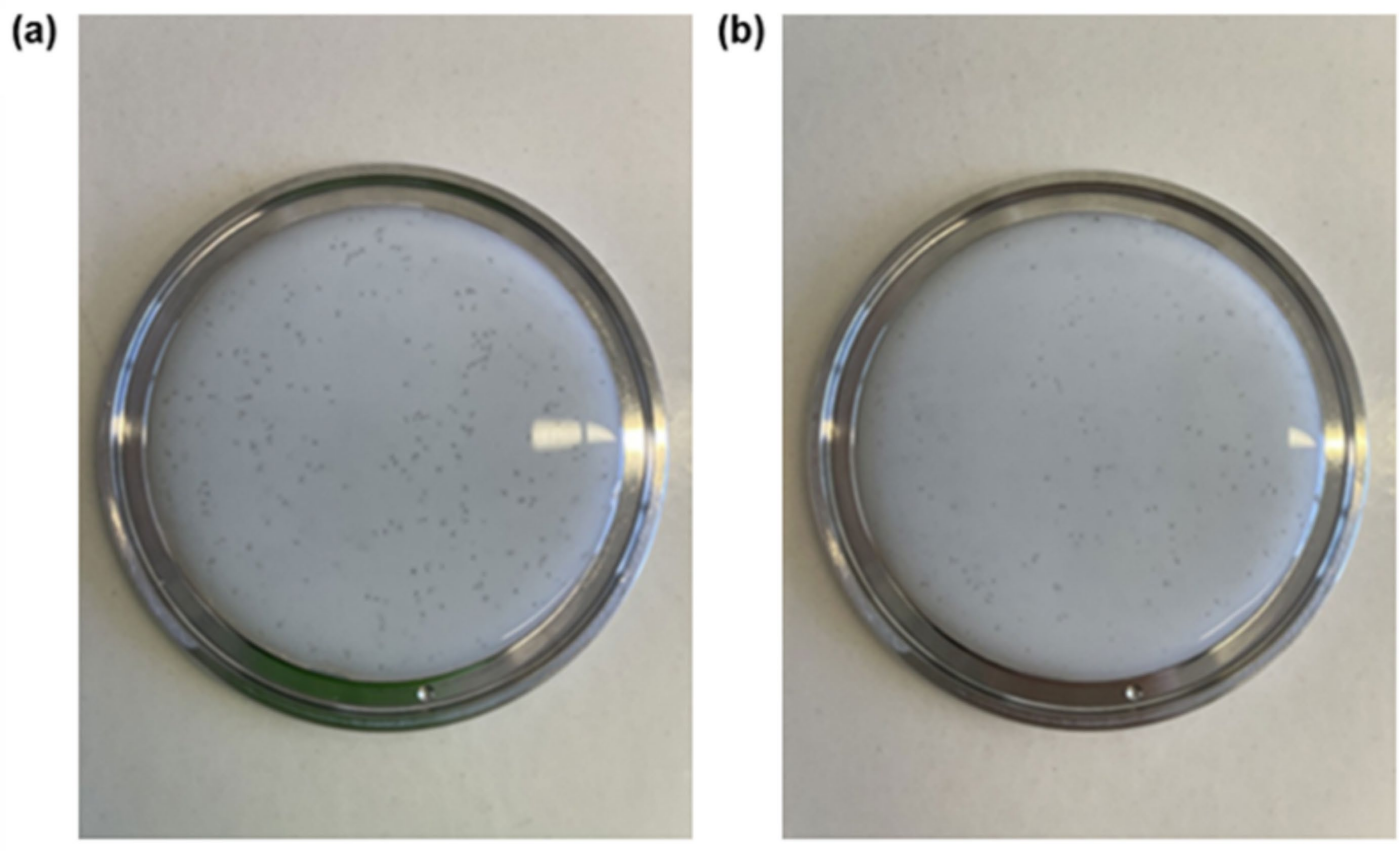
Visual representation of infant formula pre-thickened with potato starch 5 min after **(a)** preparation with moderate shaking (i.e., gently shaken by hand for approximately 20 s) or **(b)** vigorous shaking (i.e., shaken by hand back and forth twice per second for approximately 1 min at maximum intensity).

The IFPS sample was prepared using a slightly modified protocol from the supplier’s instructions. 100 mL of mineralized bottled water (Chaudfontaine®) was heated to 37 °C (± 2 °C) in a 250 mL round-bottomed flask placed in a thermostated oil bath on a magnetic stirring plate. Once the target temperature was reached, formula powder corresponding to only 50 mL of water was added under continuous stirring (approx. 300 rpm). According to the supplier, the recommended concentration is 14.333 g/100 mL. In this study, the concentration used was 7.167 g/100 mL. This 1:1 dilution (formula-to-water) was applied to allow comparison with samples prepared for the advanced gastric environment simulation, which included 50 mL of formula and 50 mL of enzymatic solution (see “advanced simulation protocol”). The IFPS samples were *vigorously* shaken by hand (i.e., shaking back and forth twice per second, which is a higher intensity than one would naturally apply in real-world conditions) for approximately 1 min, followed by magnetic stirring at 300 rpm. This methodological adjustment was based on the observation of visible graininess impairing full dissolution until between 10 and 20 min post-preparation (Figure 1).

As SFRF and IFPS samples are diluted, the results will be an underestimation of their true viscosity. Immediately after preparation, the pH of the samples was monitored using a potentiometric pH meter (HANNA HI 3320 pH/ORP Meter, HANNA instruments, Rhode Island, United States).

### 2.3 Bottle simulation protocol

Viscosity measurements (mPa*s) of SFRF and IFPS were first conducted at pH 7 and 20 °C at 10 and 20 min after sample preparation. Additional measurements were then performed at pH 7 and 37 °C at 5, 10, 20, 30, and 60 min post-preparation.

### 2.4 Simple gastric environment simulation protocol

In the simple gastric environment simulation protocol, experiments were carried out at pH 1 and pH 4 to, respectively, simulate a standard intragastric pH in infants and an intragastric pH under administration of proton pump inhibitors [PPIs] (3, 6, 10). To achieve these pH values, samples were acidified by dropwise addition of a 10 M HCl solution. pH of the samples was monitored using the previously mentioned potentiometric pH meter. Viscosity measurements of SFRF and IFPS were conducted at 37 °C at 5, 10, 20, 30, and 60 min post-preparation.

### 2.5 Advanced gastric environment simulation protocol

To analyze the viscosities of SFRF and IFPS in an advanced simulated gastric environment, artificial saliva and simulated gastric fluid were added. These solutions were prepared in a sampling protocol identical to the *in vitro* digestion flow diagram as described by Prakash et al. (6), yielding an artificial saliva/infant formula ratio of 2.069 g/100 g. Artificial saliva was prepared by adding *α*-amylase (20,000 units/100 mL, calculated with an applied ratio of 1.5 U/mg), originating from *Aspergillus oryzae*, to 15 mL of a formulated saline solution. The latter was assembled by dissolving NaHCO_3_ (0.521 g/100 mL), K_2_HPO_4_^.^3H_2_O (0.137 g/100 mL), NaCl (0.088 g/100 mL), KCl (0.048 g/100 mL) and CaCl_2_^.^2H_2_O (0.041 g/100 mL) in distilled water (11). After the addition of α-amylase, the solution was diluted in distilled water until a volume of 20 mL was reached (6). Simulated gastric fluid was prepared by adding 0.320 g/100 mL pepsin, originating from porcine gastric mucosa, to 50 mL of a 0.2% NaCl-solution (6).

Amounts of 1.244 g and 1.174 g of artificial saliva were added to 50 mL of previously prepared SFRF and IFPS respectively, adjusting for the differing densities. Subsequently, 50 mL of simulated gastric fluid was added. Viscosity measurements were then conducted at pH 7 and 37 °C at 5, 10, 20, 30, and 60 min post-preparation. Subsequently, the rheological experiments were repeated at pH 1 and pH 4 (similarly at 37 °C and 5, 10, 20, 30, and 60 min) to, respectively, simulate a standard intragastric pH in infants and an intragastric pH under administration of proton pump inhibitors [PPIs] (3, 6, 10). Acidification happened by dropwise addition of a 10 M HCl solution under pH monitoring.

### 2.6 Rheological analysis

Rheological analysis was conducted within an experimental cycle of 60 min. as this was considered a realistic representation of the timeframe during which an infant might consume a feed. Samples of ± 3.5 mL were analyzed using a stress-controlled rotational rheometer (Haake Mars III, Thermo Fisher Scientific, Waltham, United States), with flat, polished 60 mm titanium parallel plate geometry to evaluate viscosities. A Peltier temperature module ensured a controlled temperature of 37 °C in all experiments. After zero gap determination at the test temperature, samples were equilibrated at the measuring gap of 0.001 m (± 0. 25 × 10^−3^ m) for 1–2 min. prior to analysis. A rotational test with shear rates varying from 50 s^−1^ to 0.01 s^−1^ was applied in 15 equally distributed steps (logarithmically). Viscosity values determined at the single shear rate of 50 s^−1^ were reported as this shear rate is representative for the shear stresses of the digestive system (6, 12). The following steps were held for at least 15 s to ensure stabilization of the sample. Each experiment was run in triplicate for both SFRF and IFPS, three acidity levels and three preparation protocols.

### 2.7 Statistical analysis

Results were analyzed in Microsoft Excel and IBM SPSS Statistics, version 29 (IBM Corp., Armonk, N.Y., United States). A descriptive analysis was chosen, as statistical significance offers limited clinical value in this context as currently, no defined viscosity thresholds are linked to a therapeutic reduction in reflux episodes.

## 3 Results

A detailed numeric overview of the viscosities and standard deviations of SFRF and IFPS per acidity level and per simulation protocol in function of time, resulting from 204 rheological experiments, is presented as Supplementary material.

### 3.1 Optimal preparation method for milk formulas containing thickeners

**Figure 2:**
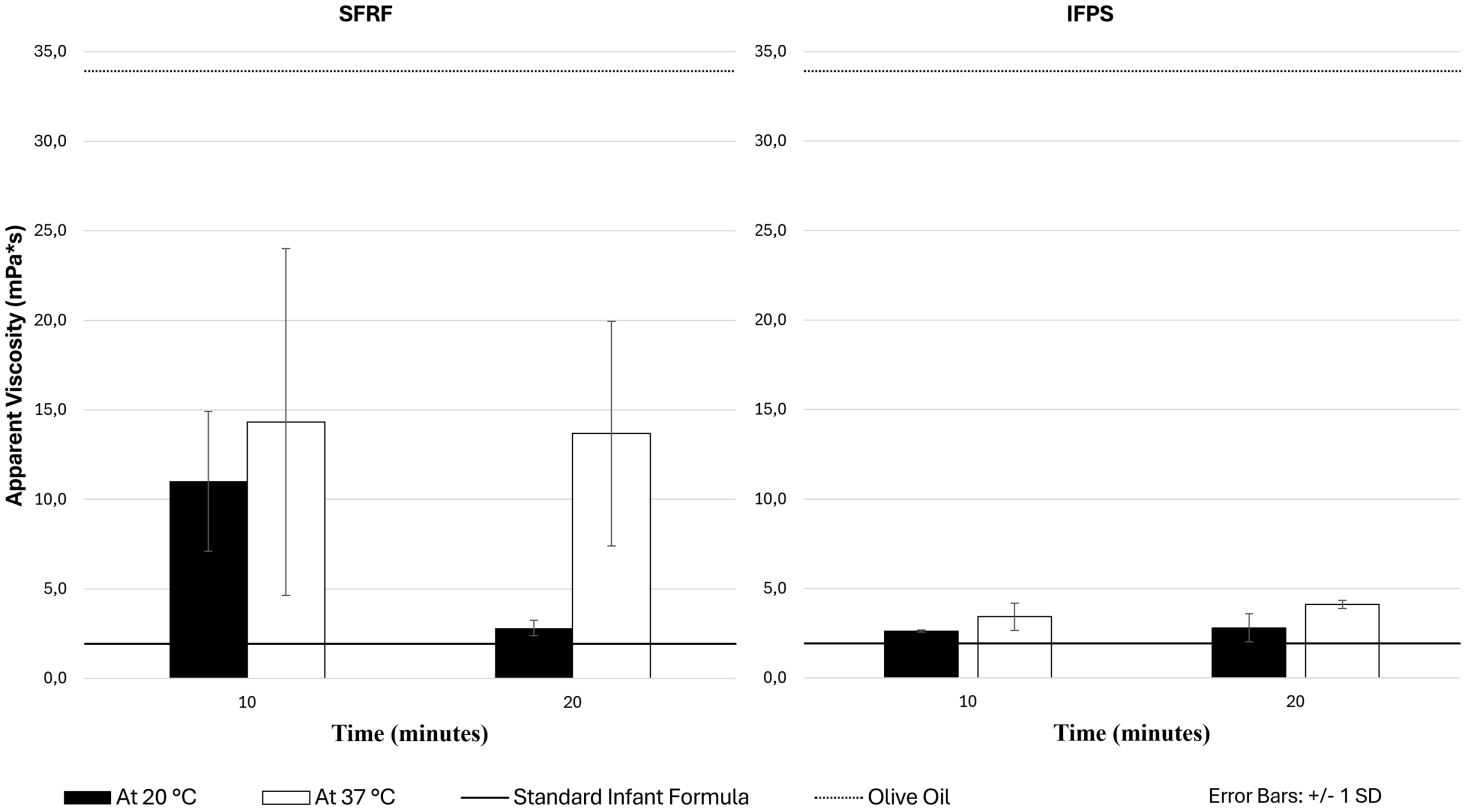
Comparison of the viscosities in function of time at room temperature (20 °C) and body temperature (37 °C). SFRF, standard infant formula thickened with rice flour. IFPS, pre-thickened infant formula with potato starch.

**Figure 3:**
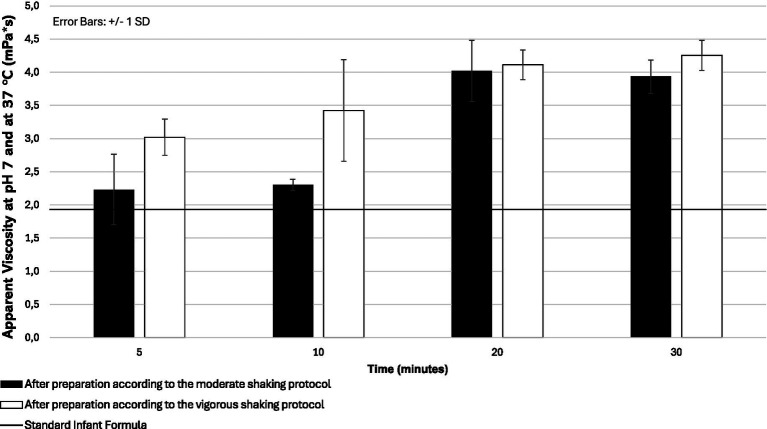
Viscosities of infant formula pre-thickened with potato starch (IFPS) under bottle simulation conditions (pH 7, 37 °C) following a moderate shaking protocol (gently shaken by hand for ~20 s) or a vigorous shaking protocol (shaken by hand at maximum intensity for ~1 min).

As shown in Figure 2 and Supplementary Table S3, both SFRF and IFPS reached higher viscosities at 37 °C compared to 20 °C at 10 (SFRF: 14.3 ± 9.7 vs. 11.0 ± 3.9 mPa*s; IFPS: 3.4 ± 0.8 vs. 2.6 ± 0.1 mPa*s) and 20 (SFRF: 13.7 ± 6.3 vs. 2.80 ± 0.4 mPa*s; IFPS: 4.1 ± 0.2 vs. 2.80 ± 0.8) minutes post-preparation, with a markedly larger effect for SFRF. Figure 3 illustrates the time-dependent viscosity of IFPS and indicates that the vigorously shaken sample reaches its peak viscosity more quickly than the moderately shaken one. Nevertheless, both methods achieve a stable final viscosity by 20 min post-preparation. Temperature and time are therefore key factors in achieving desired viscosity in infant formulas (pre-)thickened with starch.

### 3.2 Infant formula behavior in the bottle

**Figure 4:**
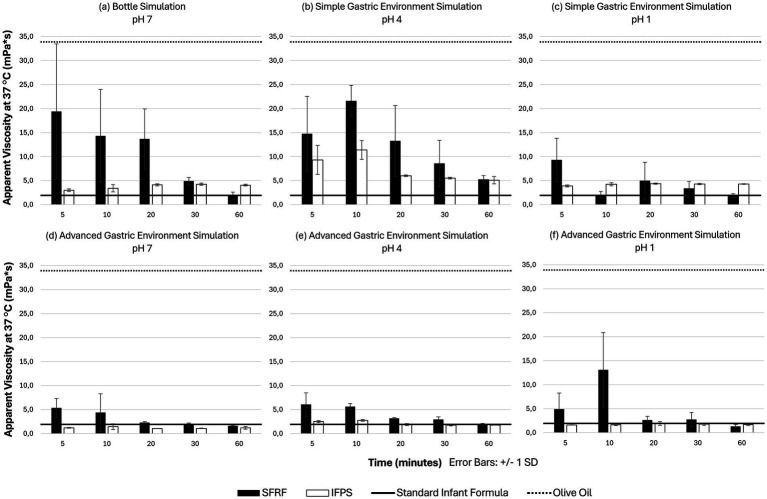
Viscosities of SFRF and IFPS, displayed at different time points and acidity levels at a single shear rate of 50 s^−1^. The upper three panels of the figure demonstrate rheological analyses of milk samples in the absence of enzymes at pH 7 **(a)**, 4 **(b)**, and 1 **(c)**. The lower three panels represent viscosities of samples to which enzymes were added with the aim of accurately simulating the gastric environment, at pH 7 (for reasons of comparison) **(d)**, pH 4 (under simulated PPI environment) **(e)**, and pH 1 (under physiological gastric environment) **(f)**. SFRF, standard infant formula thickened with rice flour. IFPS, pre-thickened infant formula with potato starch.

Figure 4a presents the viscosity profiles of SFRF and IFPS prepared at 37 °C. SFRF reaches its peak viscosity within 5 min but gradually thins over time, with a marked decline between 20 and 30 min. In contrast, IFPS—when vigorously shaken—achieves its maximum viscosity around 20 min post-preparation and remains relatively stable thereafter.

During the first 20 min, SFRF exhibits substantially higher viscosities than IFPS [SFRF: 19.4 ± 14.1 mPa*s (5 min.); 13.7 ± 6.3 mPa*s (20 min.); IFPS: 3.0 ± 0.3 mPa*s (5 min.); 4.1 ± 0.2 mPa*s (20 min.)], despite comparable starch concentrations (2.000 g/100 mL for SFRF vs. 2.500 g/100 mL for IFPS). However, this difference diminishes by 30 min (SFRF: 4.9 ± 0.8 mPa*s vs. IFPS: 4.3 ± 0.2 mPa*s) and reverses after 1 h at room temperature, with IFPS ultimately showing higher viscosity (SFRF: 1.9 ± 0.7 mPa*s vs. IFPS: 4.1 ± 0.2 mPa*s).

### 3.3 Infant formula behavior in a simple simulated gastric environment

Figure 4b shows that acidifying SFRF to pH 4 initially increases viscosity at the 10-min time point, followed by a sharp decline over time [SFRF: 21.6 ± 3.2 mPa*s (10 min.); 13.3 ± 7.4 mPa*s (20 min.); 5.3 ± 0.8 mPa*s (60 min.)]. Further reducing the pH to 1 (Figure 4c) results in consistently low viscosities across all time points. IFPS demonstrates a viscosity increase when the pH is lowered from 7 to 4 [e.g., IFPS at 10 min.: 3.4 ± 0.8 mPa*s (pH 7); 11.4 ± 2.0 mPa*s (pH 4); Figure 4b], but no further increase is observed at pH 1 (Figure 4c), with values remaining similar to those at pH 7 throughout the evaluation period [IFPS at 10 min.: 4.3 ± 0.3 mPa*s (pH 1)].

### 3.4 Infant formula behavior in an advanced simulated gastric environment

Figure 4d is a reference panel allowing for a methodological assessment of enzymatic hydrolysis in interaction with different acidity levels. Figure 4e corresponds to the advanced simulated gastric environment with the use of PPI’s, i.e., 37 °C, pH 4 (3, 6, 10, 13). Figure 4f illustrates the mean viscosities of SFRF and IFPS in a physiological gastric environment (37 °C, pH 1).

In the advanced simulated gastric environment—both under conditions mimicking PPI use (pH 4) and normal physiological acidity (pH 1)—the viscosities of both SFRF and IFPS closely resembled those of the SF without any thickening agents (e.g., SFRF at pH 1, 20 min.: 2.6 ± 0.8 mPa*s; IFPS at pH 1, 20 min.: 1.9 ± 0.4 mPa*s vs. SF at pH 7, without enzymes: 2.0 ± 0.1 mPa*s). Notably, when compared to their performance in the bottle, both SFRF and IFPS lost nearly all viscosity-enhancing capacity between 10 and 20 min of exposure to the simulated gastric conditions.

### 3.5 The daily added caloric value due to starch thickeners

Table 2 shows that the addition of calorie-dense, starch-based thickeners to infant formula can significantly increase an infant’s daily energy intake. Assuming a daily consumption of 150 mL/kg of SFRF, infants weighing between 3 and 6 kg may exceed their recommended daily energy requirements by 16 to 66%. Notably, the rice flour alone can contribute up to 37% of the total daily energy need.

**Table 2:** Excess energy intake of infants consuming standard infant formula thickened with rice flour [SFRF] when a daily intake of 150 mL milk/kg is assumed.

Weight (kg)		3	4	5	6
Average daily milk intake (mL)		450	600	750	900
Average daily energy intake from milk^1^ (kcal)		301.5	402	502.5	603
Average additional daily energy intake supplied by rice flour^2^ (kcal)		86.6	115.5	144.4	173.3
Total average daily energy intake supplied by SFRF (kcal)		388.1	517.5	646.9	776.3
Adequate daily energy intake (kcal)	AMAB AFAB	327 309	448 428	475 460	468 474
Percentage of excess daily energy intake, per sex	AMAB AFAB	19% 26%	16% 21%	36% 41%	66% 64%
Percentage of adequate energy intake supplied by rice flour, per sex	AMAB AFAB	26% 28%	26% 27%	30% 31%	37% 37%

AFAB, Assigned Female At Birth; AMAB, Assigned Male At Birth.

1 Assuming a caloric density of 67 kcal/100 mL for milk.

2 The recommended instructions for use are to add 5 gram (= 19.25 kcal) of rice flour to 100 mL of infant formula.

## 4 Discussion

The study findings indicate that infant formulas containing starch or supplemented with starch-based thickeners must be shaken vigorously to achieve proper solubilization and are preferably prepared at temperatures higher than room temperature. Additionally, standard formulas thickened with rice flour seem to be unstable (i.e., drastic decrease in viscosity) at room temperature beyond 20 min., making it advisable to prepare them immediately before consumption. Infant formulas pre-thickened with potato starch, on the other hand, only reach their highest viscosity at 30 min. However, simulated gastric conditions suggest that the increased viscosity observed in the bottle does not persist in the gastric environment and—under those conditions—do not exceed the viscosity of a standard formula without thickening properties. This raises questions about their clinical applicability. Lastly, the addition of a starch-based thickener significantly increases an infant’s consumption of calories, causing an excess energy intake.

Four determinators can affect the viscosities in infant formula thickened with starches: acid hydrolysis, enzymatic hydrolysis, the whey/casein ratio of infant formulas and the amylose content of the thickening agent. Firstly, starches are prone to acid hydrolysis, leading to a decrease in viscosity at low pH (14). On the contrary, adding only a small amount of acid promotes leaching out of amylose chains, having a viscosity increasing effect. This process takes place when starches are heated in water and their granules swell, forming hydrogen bonds in the amorphous regions of the granules and subsequently transmitting disruptive forces to the crystalline regions (14–17). Hence, the peak viscosity at 10 min. (Figure 4b—simple gastric simulation at pH 4) might be due to an optimal environment for amylose chains to leach out while acid hydrolysis is not yet dominant. It is known that potato starch behaves more resistantly against acid hydrolysis when compared to rice flour, which may explain the finding that IFPS shows more stable viscosities across the three acidity levels (16, 18).

It should be noted that both starches used as thickener for infant formula are pregelatinized, implying that they were pre-cooked in an excess of shear and water. This gelatinization step enables subsequent thickening of starch at relatively low temperatures due to disruption of the initial starch granules. However, pregelatinized potato- and rice-based starches exhibit different physicochemical properties upon dispersion which can mainly be attributed to differences in amylose content. According to Xie et al., the increased viscosity associated with a higher amylose content is explained by the unique microstructure and phase transitions of starch as the highly branched structure of amylopectin and the formation of *gel-balls* and *super-globes* during gelatinization result in less polymer chain entanglement in amylopectin-rich starch compared to amylose-rich linear starch (19). Additionally, amylose and amylopectin behave differently upon retrogradation. Starches richer in amylose retrograde faster, which might also contribute to the higher viscosities reached in SFRF (20).

Considering temperature, SFRF shows markedly higher viscosities at body temperature (37 °C) as compared to room temperature (20 °C), which aligns with the physicochemical properties of especially rice starch. It is known that both the swelling power and water solubility of starches tend to increase with a corresponding increase in temperature (20). The more pronounced effect in SFRF compared to IFPS can be attributed to a higher amylose content and lower crystalline fraction of rice starch compared to potato starch resulting more swelling at lower temperatures (15, 19, 20). Our findings suggest that when relying on thickeners to slow down the flow through the teat and thus lengthening the feeding time, it is advisable to prepare thickened formula, especially SFRF, at body temperature to maximize the thickening at a given concentration.

Considering stand time of prepared bottles, guidelines differ per country. This inconsistency can likely be attributed to the variety of (pre-)thickened infant formulas that are studied (3, 7, 21, 22). Our results suggest that the required stand time for maximal in-bottle viscosity varies by the type of starch-based thickener used. Potato starch in IFPS requires approximately 30 min of standing time to reach maximal viscous capacity while rice flour in SFRF reaches its peak viscosity 5 min after preparation and decreases in viscosity over time. This finding is analogous to the results of a study where samples thickened with rice cereal were significantly thinner after 30 min of stand time as compared with 5 min of stand time (23). To date, thickening agents come with standard instructions that do not take into account the individual makeup of the infant formula with which they will be mixed (23). Based on our *in vitro* experiments, a two-part recommendation can be deduced: (1) infant formula that is externally thickened with rice flour, requires a stand time as short as possible, whereas (2) commercially available formula pre-thickened with potato starch should be administered half an hour post-preparation.

The process of shaking plays a critical role in the preparation of IFPS. The manufacturer emphasizes this by clearly instructing using bold and underlined text on the packaging that the powder must be shaken *vigorously*. In our study, we found that the clinical application of this instruction corresponds with manual shaking it back and forth twice every second, for 1 min. This is necessary to ensure proper solubilization of starch granules. Therefore, this technique was considered the best-fit empirical interpretation of the manufacturer’s instruction. However, this technique may introduce air bubbles into the formula, contributing to reflux or aspiration (5, 24). Overall, the evidence supporting the use of thickened infant formulas as a strategy to reduce aspiration in infants remains limited (25).

When the goal is to achieve a high viscosity directly in the bottle, the findings of the current study suggest that SFRF is preferable to IFPS, as it can reach viscosities up to 6 times higher under comparable conditions. This makes SFRF potentially more effective for managing conditions requiring high bottle viscosity. This could be explained by the presence of an on average higher amylose content in rice flour (± 20%) compared to potato starch (11.9–20.1%) (26, 27). A key limitation appears however to be the stability of SFRF: its viscosity begins to decline noticeably after 20 min post-preparation. This finding supports that consumption within the 20 min following bottle preparation is an advisable feeding strategy. Delayed feeding may result in reduced viscosity, potentially diminishing its clinical effectiveness and altering feeding performance.

On the other hand, as excessively thickened infant formula may demand a highly increased sucking effort during the feed, care should be taken in avoiding fatigue, decreased nutritional intake and subsequent undernutrition and dehydration (23). The viscosities reported in this study imply that thickened infant formulas require faster flowing bottle nipples to accommodate the fluid consistency, unless a slow flow is aimed at. It should be taken into consideration that thickened formulas may require a tailored teat size when adopting or switching between thickening strategies (5, 21, 23, 28, 29).

In the advanced simulated gastric environment, which includes enzymes and an acidic setting, viscosities at pH 7 were approximately three times lower than in the bottle simulation, likely due to the immediate enzymatic hydrolysis of both the protein and the starch in the infant formulas. The outlier viscosity observed in Figure 4f may result from the strong acidity (pH 1) inhibiting *α*-amylase activity (6). Differences between the two milk formulas in terms of impact of acidity and time may be attributed to variations in whey/casein ratio: 50/50 in SFRF and 70/30 in IFPS. The higher casein fraction in SFRF may account for its generally more viscous behavior compared to IFPS in a mildly acidic environment. The rheological results support the presence of two viscosity-enhancing mechanisms in SFRF: acid-induced aggregation of casein and pepsin-induced coagulation of the casein fraction in the stomach (14, 30). These mechanisms also align with the outlier viscosity observed in SFRF in the gastric environment (Figure 4f). Our results confirm the physiological effects of gastric coagulation in casein-rich milk formula, enabling to slow down the gastric emptying process (14, 31).

It needs to be addressed that the viscosities of both SFRF and IFPS under simulated gastric conditions closely resemble those of a standard formula without any thickening agents. This suggests that, regardless of the gastric pH, the thickening effect of these starch-based formulas is not sustained in the stomach-like environment. Compared to the viscosity in the bottle, both SFRF and IFPS lost nearly all thickening within the first 10 min of exposure to the simulated gastric conditions. These findings raise important questions about the *in vivo* effectiveness of starch-based thickeners in reducing regurgitation or reflux symptoms.

This study highlights the potential for excess energy intake when externally adding starch-based thickeners, such as rice flour, to infant formula. Table 2 confirms existing concerns about the impact on weight gain associated with these practices, as previously noted in the literature (1, 4, 5). By calculating the additional caloric contribution of rice flour to an infant’s daily intake, our findings objectively support those of Horvath et al., who reported a statistically significant increase in weight gain with starch-thickened formulas (4). However, even in cases of undernutrition or when weight gain is desired, simply increasing the carbohydrate content of a milk feed is not an appropriate strategy (23). A balanced macronutrient profile is essential for healthy growth and development. Furthermore, the long-term metabolic consequences of routinely adding excess carbohydrates to infant feeds remain unknown, raising questions about the safety and appropriateness of this approach in clinical practice (32, 33).

The risks of aspiration when shaking IFPS bottles *vigorously* must be weighed up against the clinical disadvantage of a less viscous feed when shaking *moderately*. After all, safe thickening should be prioritized over meeting viscosity benchmarks. Correct prescriber-led counseling of parents forms a central pillar of this anti-reflux feeding strategy. Pharmacists and midwives on the other hand can play an active role in ensuring tailored feeding equipment and teat sizes. Our findings can provide a framework for healthcare professionals when educating parents on bottle stand time and shaking. Indeed, it has been shown that questions about infant formula are very prevalent in primary care (34, 35). Eventually, studies on the safety profile and clinical effectiveness of starch-based thickening are warranted. More evidence of these aspects needs to facilitate personalized decisions whether the risks of caloric overdosage, aspiration and energy expenditure during a feed outweigh the possible benefits of increased viscosity (4, 23).

This study’s strengths include the use of a rheometer as it is the recommended and user-independent method for assessing thickened fluid properties. However, the absence of specific viscosity data for therapeutic effectiveness in GORD and dysphagia limits comparability with therapeutic standards. As well, this is only the second rheological study—after Prakash, Ma (6)—that adds artificial saliva and simulated gastric fluid to formula and the first one that critically reviews the clinical effectiveness of the yielded viscosities. Another strength is that our results provide a framework for precise preparation and timing guidelines in clinical practice. Additionally, our research into the caloric impact of the addition of starch to a standard formula is the first of its kind.

A limitation of this study is the inclusion of merely two starch-based thickeners. The observed preparation challenges may not apply to all starch-based thickeners, highlighting the need for including a broader spectrum of formulas with subtle differences in amylose content and whey-casein ratios. We also did not compare different concentrations of starch-based thickeners. However, the findings from the advanced gastric acid simulation suggest that varying concentrations are unlikely to alter their effect in the stomach but they could influence viscosity in the bottle. Another limitation is the lack of comparison with established viscosity standards, such as IDDSI; however, the added value of such a comparison is uncertain, as these frameworks are not validated for infant feeding and no clinically relevant viscosity thresholds for reflux or regurgitation have been defined in this population. Third, there is a need for thoughtful translation of our *in vitro* results to the *in vivo* conditions. Our gastric simulation protocol may not fully reflect in vivo infant conditions. The gastric muscle contractions were not simulated, although they may influence casein coagulation. The model also assumes static acidity and immediate, complete enzyme exposure, whereas in reality gastric acidification and enzymatic digestion occur progressively, with gradual protein hydrolysis. Finally, amylase activity in infants under 6 months is not fully developed, reaching only about two-thirds of adult levels at 3 months, which may further affect starch digestion (14, 36). Fourth, since the current study focuses on infant formula designed for term infants, cautiousness in translating the described thickening strategies to preterm infants and to human expressed breast milk is recommended (3, 10). Salivary amylase is negligible in infants for the first 3 months post birth, whereas a highly active amylase concentration in breastmilk results in less viscous liquids that are not comparable to equivalent scenarios where thickening is performed on infant formulas (10, 29).

## 5 Conclusion

This study highlights several important challenges associated with the use of (pre-)thickened infant formulas with starch. Effective thickening depends on specific preparation conditions, including adequate temperature and standing time, the latter varying by the type of starch used. Inadequate preparation may lead to incomplete dispersion of starch granules, potentially impairing the intended viscosity. Under simulated gastric conditions—whether mimicking physiological or acid-reduced environments—starch-thickened formulas exhibited viscosities comparable to standard, non-thickened formulas. Our findings suggest that starch may be unsuitable as a thickening agent when the goal is to maintain increased viscosity in the gastric environment.

## Data Availability

The original contributions presented in the study are included in the article/Supplementary material, further inquiries can be directed to the corresponding author.

## References

[1] Haiden N Savino F Hill S Kivelä L De Koning B Kӧglmeier J . Infant formulas for the treatment of functional gastrointestinal disorders: a position paper of the ESPGHAN nutrition committee. J Pediatr Gastroenterol Nutr. (2024) 79:168–80. doi: 10.1002/jpn3.12240, 38766683

[2] Corvaglia L Spizzichino M Aceti A Legnani E Mariani E Martini S . A thickened formula does not reduce apneas related to gastroesophageal reflux in preterm infants. Neonatology. (2013) 103:98–102. doi: 10.1159/000342703 23172040

[3] Baert K Ombecq M Van Winckel M Henry S Tommelein E Vanhoorne V. The viscosity-enhancing effect of carob bean gum and sodium carboxymethylcellulose when added to infant formula. Food Sci Nutr. (2024) 12:2661–70. doi: 10.1002/fsn3.3947, 38628187 PMC11016439

[4] Horvath A Dziechciarz P Szajewska H. The effect of thickened-feed interventions on gastroesophageal reflux in infants: systematic review and meta-analysis of randomized, controlled trials. Pediatrics. (2008) 122:e1268–77. doi: 10.1542/peds.2008-1900 19001038

[5] Pados BF Mellon M. Effect of thickening on flow rates through bottle nipples. J Obstet Gynecol Neonatal Nurs. (2021) 50:78–87. doi: 10.1016/j.jogn.2020.09.153, 33223485

[6] Prakash S Ma Q Bhandari B. Rheological behaviour of selected commercially available baby formulas in simulated human digestive system. Food Res Int. (2014) 64:889–95. doi: 10.1016/j.foodres.2014.08.028, 30011729

[7] Tommelein E Baert K Ombecq M Henry S Vanhoorne V. Anti-regurgitation infant formulas and antacid medication: match or mismatch? Eur J Pediatr. (2025) 184:336. doi: 10.1007/s00431-025-06161-1 40355562

[8] Haschke F Haiden N Thakkar SK. Nutritive and bioactive proteins in breastmilk. Ann Nutr Metab. (2016) 69:16–26. doi: 10.1159/000452820 28103610

[9] Ballard O Morrow AL. Human milk composition: nutrients and bioactive factors. Pediatr Clin N Am. (2013) 60:49–74. doi: 10.1016/j.pcl.2012.10.002, 23178060 PMC3586783

[10] Corvaglia L Martini S Aceti A Arcuri S Rossini R Faldella G. Nonpharmacological management of gastroesophageal reflux in preterm infants. Biomed Res Int. (2013) 2013:141967. doi: 10.1155/2013/141967 24073393 PMC3773993

[11] Hong JH Duncan SE Dietrich AM O'Keefe SF. Effect of copper on the volatility of aroma compounds in a model mouth system. J Agric Food Chem. (2006) 54:9168–75. doi: 10.1021/jf061229m, 17117806

[12] Cichero J Nicholson T Dodrill P. Liquid barium is not representative of infant formula: characterisation of rheological and material properties. Dysphagia. (2011) 26:264–71. doi: 10.1007/s00455-010-9303-3, 20830598

[13] Safe M Chan WH Leach ST Sutton L Lui K Krishnan U. Widespread use of gastric acid inhibitors in infants: are they needed? Are they safe? World J Gastrointest Pharmacol Ther. (2016) 7:531–9. doi: 10.4292/wjgpt.v7.i4.531, 27867686 PMC5095572

[14] Huppertz T Chia LW. Milk protein coagulation under gastric conditions: a review. Int Dairy J. (2021) 113:104882. doi: 10.1016/j.idairyj.2020.104882

[15] Biduski B Silva W Colussi R Halal S Lim LT Dias ÁRG . Starch hydrogels: the influence of the amylose content and gelatinization method. Int J Biol Macromol. (2018) 113:443–9. doi: 10.1016/j.ijbiomac.2018.02.144, 29486261

[16] Hirashima M Takahashi R Nishinari K. Effects of adding acids before and after gelatinization on the viscoelasticity of cornstarch pastes. Food Hydrocoll. (2005) 19:909–14. doi: 10.1016/j.foodhyd.2004.12.004

[17] Man J Qin F Zhu L Shi YC Gu M Liu Q . Ordered structure and thermal property of acid-modified high-amylose rice starch. Food Chem. (2012) 134:2242–8. doi: 10.1016/j.foodchem.2012.04.100, 23442680

[18] Salvatore S Savino F Singendonk M Tabbers M Benninga MA Staiano A . Thickened infant formula: what to know. Nutrition. (2018) 49:51–6. doi: 10.1016/j.nut.2017.10.010, 29495000

[19] Xie F Yu L Su B Liu P Wang J Liu H . Rheological properties of starches with different amylose/amylopectin ratios. J Cereal Sci. (2009) 49:371–7. doi: 10.1016/j.jcs.2009.01.002

[20] Zhu F Xie Q. "Structure and physicochemical properties of starch". In: Sui Z Kong X, editors. Physical Modifications of Starch. Singapore: Springer Singapore (2018). p. 1–14.

[21] Cichero JA Nicholson TM September C. Thickened milk for the management of feeding and swallowing issues in infants: a call for interdisciplinary professional guidelines. J Hum Lact. (2013) 29:132–5. doi: 10.1177/0890334413480561, 23507962

[22] Gosa MM Choquette CK. Effect of commercially available thickening agents on ready-to-feed infant formulas. J Texture Stud. (2021) 52:612–22. doi: 10.1111/jtxs.12600 33843078

[23] Gosa MM Dodrill P. Effect of time and temperature on thickened infant formula. Nutr Clin Pract. (2017) 32:238–44. doi: 10.1177/0884533616662991, 27581202

[24] Marshall J Buttsworth J Grandt HDS Raatz M Signorini A Fernando S . Testing and development of slightly thick infant formula recipes for dysphagia management: an Australian perspective. Dysphagia. (2023) 38:1254–63. doi: 10.1007/s00455-022-10550-1, 36637506 PMC10326089

[25] Gosa M Schooling T Coleman J. Thickened liquids as a treatment for children with dysphagia and associated adverse effects: a systematic review. ICAN: Infant, Child, & Adolescent Nutrition. (2011) 3:344–50. doi: 10.1177/1941406411407664

[26] Farooq MA Murtaza MA Aadil RM Arshad R Rahaman A Siddique R . Investigating the structural properties and in vitro digestion of rice flours. Food Sci Nutr. (2021) 9:2668–75. doi: 10.1002/fsn3.2225, 34026080 PMC8116841

[27] Talja RA Peura M Serimaa R Jouppila K. Effect of amylose content on physical and mechanical properties of potato-starch-based edible films. Biomacromolecules. (2008) 9:658–63. doi: 10.1021/bm700654h, 18166015

[28] Pados BF Davitt ES. Pathophysiology of gastroesophageal reflux disease in infants and nonpharmacologic strategies for symptom management. Nurs Womens Health. (2020) 24:101–14. doi: 10.1016/j.nwh.2020.01.005, 32101759

[29] Pados BF Park J Thoyre SM Estrem H Nix WB. Milk flow rates from bottle nipples used for feeding infants who are hospitalized. Am J Speech Lang Pathol. (2015) 24:671–9. doi: 10.1044/2015_AJSLP-15-0011, 26172340 PMC4698468

[30] Phosanam A Chandrapala J Huppertz T Adhikari B Zisu B. In vitro digestion of infant formula model systems: influence of casein to whey protein ratio. Int Dairy J. (2021) 117:105008. doi: 10.1016/j.idairyj.2021.105008

[31] Moukarzel AA Abdelnour H Akatcherian C. Effects of a prethickened formula on esophageal pH and gastric emptying of infants with GER. J Clin Gastroenterol. (2007) 41:823–9. doi: 10.1097/MCG.0b013e31802c2a10, 17881928

[32] Cheung KY Petrou L Helfer B Porubayeva E Dolgikh E Ali S . Health and nutrition claims for infant formula: international cross sectional survey. BMJ. (2023) 380:e071075. doi: 10.1136/bmj-2022-071075, 36792145 PMC9930154

[33] Bridge G Lomazzi M Bedi R. A cross-country exploratory study to investigate the labelling, energy, carbohydrate and sugar content of formula milk products marketed for infants. Br Dent J. (2020) 228:198–212. doi: 10.1038/s41415-020-1252-0, 32060463

[34] Tommelein E De Boevre M Vanhie L Van Tongelen I Boussery K De Saeger S. Revisiting the food- and nutrition-related curriculum in healthcare education: an example for pharmacy education. Pharmacy. (2021) 9:104. doi: 10.3390/pharmacy9020104, 34067396 PMC8162543

[35] Gorsen S Boussery K Van Winckel M Demeyer R Tommelein E. Perspectives of parents and health care providers about (non)medical treatment in infants with reflux. Pharmacy. (2020) 8:226. doi: 10.3390/pharmacy8040226, 33238424 PMC7712772

[36] Sevenhuysen GP Holodinsky C Dawes C. Development of salivary alpha-amylase in infants from birth to 5 months. Am J Clin Nutr. (1984) 39:584–8. 6608871 10.1093/ajcn/39.4.584

