# Supplementary material for: Starch-based thickening in infant formula: in vitro study of behavior in the bottle and under gastric conditions
Source: Front Nutr. 2026 Apr 10;13:1803756. doi: 10.3389/fnut.2026.1803756 (PMC13106065; doi:10.3389/fnut.2026.1803756)
Supplement: Supplementary file 1 [file Table_1.docx]

| **Table S1:** Overview of the mean apparent viscosities (mPa*s) and standard deviations resulting from a triplicate of rheological analyses.  **Bottle simulation:** infant formula preparation protocol per 100 mL of mineralized bottled water (Chaudfontaine^®^) without the addition of enzymes.  **Simple gastric environment simulation:** infant formula preparation protocol similar to bottle simulation but followed by pH-adjustments.  **Advanced gastric environment simulation:** infant formula preparation protocol using a 1/1 ratio of mineralized bottled water and simulated enzymatic solution, yielding an artificial saliva/infant formula ratio of 2.1 g/100 g. | | | | | | |
| --- | --- | --- | --- | --- | --- | --- |
| **SFRF**: **Apparent viscosity (mPa*s) of** samples of standard formula thickened with rice flour with a starch-based thickener fraction of 2.5 g/100 mL. | | | | | | |
| **SFRF viscosity**  Mean (SD) in mPa·s | **pH 7** | | **pH 4** | | **pH 1** | |
|  | **Bottle simulation** | **Advanced gastric simulation** | **Simple gastric simulation** | **Advanced gastric simulation** | **Simple gastric simulation** | **Advanced gastric simulation** |
| **5min.** | 19.4 (14.1) | 5.3 (2.0) | 14.8 (7.8) | 6.0 (2.4) | 9.3 (4.5) | 4.9 (3.3) |
| **10min.** | 14.3 (9.7) | 4.4 (3.9) | 21.6 (3.2) | 5.6 (0.6) | 2.1 (0.7) | 13.1 (7.8) |
| **20min.** | 13.7 (6.3) | 2.3 (0.2) | 13.3 (7.4) | 3.2 (0.1) | 5.0 (3.8) | 2.6 (0.8) |
| **30min.** | 4.9 (0.8) | 1.8 (0.4) | 8.6 (4.8) | 2.9 (0.6) | 3.4 (1.4) | 2.8 (1.5) |
| **60min.** | 1.9 (0.7) | 1.6 (0.1) | 5.3 (0.8) | 1.9 (0.3) | 1.9 (0.4) | 1.3 (0.4) |
