# Supplementary material for: Starch-based thickening in infant formula: in vitro study of behavior in the bottle and under gastric conditions
Source: Front Nutr. 2026 Apr 10;13:1803756. doi: 10.3389/fnut.2026.1803756 (PMC13106065; doi:10.3389/fnut.2026.1803756)
Supplement: Supplementary file 2 [file Table_2.docx]

| **Table S2: IFPS**: **Apparent viscosity (mPa*s) of** infant formula samples pre-thickened with potato starch with a starch-based thickener fraction of 2 g/100 mL. | | | | | | |
| --- | --- | --- | --- | --- | --- | --- |
| **IFPS viscosity**  Mean (SD) in mPa·s | **pH 7** | | **pH 4** | | **pH 1** | |
|  | **Bottle simulation** | **Advanced gastric simulation** | **Simple gastric simulation** | **Advanced gastric simulation** | **Simple gastric simulation** | **Advanced gastric simulation** |
| **5min.** | 3.0 (0.3) | 1.2 (0.1) | 9.3 (3.0) | 2.5 (0.2) | 3.9 (0.2) | 1.6 (0.0) |
| **10min.** | 3.4 (0.8) | 1.5 (0.6) | 11.4 (2.0) | 2.7 (0.2) | 4.3 (0.3) | 1.7 (0.2) |
| **20min.** | 4.1 (0.2) | 1.1 (0.0) | 6.0 (0.2) | 1.9 (0.2) | 4.4 (0.1) | 1.9 (0.4) |
| **30min.** | 4.3 (0.2) | 1.1 (0.0) | 5.5 (0.2) | 1.8 (0.2) | 4.3 (0.1) | 1.8 (0.2) |
| **60min.** | 4.1 (0.2) | 1.2 (0.3) | 5.1 (0.8) | 1.8 (0.0) | 4.3 (0.1) | 1.7 (0.2) |
