# Supplementary material for: Starch-based thickening in infant formula: in vitro study of behavior in the bottle and under gastric conditions
Source: Front Nutr. 2026 Apr 10;13:1803756. doi: 10.3389/fnut.2026.1803756 (PMC13106065; doi:10.3389/fnut.2026.1803756)
Supplement: Supplementary file 3 [file Table_3.docx]

| **Table S3:** Comparison of the impact of temperature on the apparent viscosities (mPa*s) of two infant formulas analysed at pH 7. | | | | |
| --- | --- | --- | --- | --- |
| **Bottle simulation viscosity** Mean (SD) | **SFRF** | | **IFPS** | |
|  | **20°C** | **37°C** | **20°C** | **37°C** |
| **10min.** | 11.0 (3.9) | 14.3 (9.7) | 2.6 (0.1) | 3.4 (0.8) |
| **20min.** | 2.8 (0.4) | 13.7 (6.3) | 2.8 (0.8) | 4.1 (0.2) |
